# Ultrafine α‐Phase Molybdenum Carbide Decorated with Platinum Nanoparticles for Efficient Hydrogen Production in Acidic and Alkaline Media

**DOI:** 10.1002/advs.201802135

**Published:** 2019-02-21

**Authors:** Hee Jo Song, Myeong‐Chang Sung, Hyunseok Yoon, Bobae Ju, Dong‐Wan Kim

**Affiliations:** ^1^ School of Civil Environmental and Architectural Engineering Korea University Seoul 02841 South Korea

**Keywords:** electrocatalysts, hydrogen evolution reaction, nanoparticles, platinum, α‐MoC_1−_*_x_*

## Abstract

The development of efficient electrocatalysts is important to produce clean and sustainable hydrogen fuel on a large scale. With respect to cathodic reactions, Pt exhibits an overwhelming electrocatalytic capability in the hydrogen evolution reaction (HER) in comparison with other earth‐abundant electrocatalysts, despite its rarity and high cost. So, a hybrid catalyst that combines a low‐cost electrocatalyst with Pt would balance cost‐effectiveness with catalytic activity. Herein, α‐phase molybdenum carbide (MoC_1−_ *_x_*) nanoparticles (NPs) decorated with a small amount of Pt (MoC_1−_ *_x_*/Pt‐NPs) are designed to achieve high‐performance hydrogen production in acidic and alkaline media. MoC_1−_ *_x_*‐NPs exhibit good electrocatalytic HER activity as well as stability and durability. They show favorable catalytic kinetics in an alkaline medium, suggesting an active water dissociation process. After Pt decoration, Pt‐NPs that are 2–3 nm in diameter are well incorporated with MoC_1−_ *_x_*‐NPs. MoC_1−_ *_x_*/Pt‐NPs with a small amount of Pt (2.7–3 wt%) and are able to perform superior electrocatalytic HER activity, and possess stability and durability that is comparable to that of commercial Pt/C. Notably, they exhibit a higher intrinsic catalytic activity compared to that of Pt/C in an alkaline medium, indicating that they promote the sluggish catalytic kinetics of Pt in alkaline medium.

Hydrogen molecule (H_2_) is one of the most promising fuels because it is a clean and sustainable source of energy and can replace fossil fuels.^1^, ^2^, ^3^, ^4^ For the sustainable, environment friendly, and large‐scale production of H_2_, electrochemical water splitting driven by sunlight has been considered as an attractive method.^1^, ^5^ In water electrolysis, it is required to high‐performance electrocatalysts with high catalytic activities and kinetics to promote the hydrogen evolution reaction (HER). From the viewpoint of cathodic reactions, platinum (Pt) has been identified as the most effective HER electrocatalyst but its high cost, which is due to its scarcity, hinders its applicability in large‐scale electrolysis.^6^ Despite the efforts made to the development of more abundant HER electrocatalysts, including chalcogenides,^7^, ^8^, ^9^ carbides,^10^, ^11^ phosphides,^6^, ^12^ metal/alloys,^13^, ^14^, ^15^ and carbon nitride,^16^ Pt‐based materials continue to possess unparalleled activity compared to earth‐abundant electrocatalysts.^17^

In this regard, the hybrid catalysts that combine low‐cost catalysts with a small amount of Pt can provide the active and stable electrocatalytic performance toward HER, especially in alkaline medium by promoting the sluggish catalytic kinetics of Pt such as MoS_2_/Pt,^18^ Ni_3_N/Pt,^19^ and CoS_2_/Pt.^20^ Molybdenum carbides have been considered as HER electrocatalysts owing to their excellent catalytic activities, which originate from unique Pt‐like d‐band electronic structures,^1^, ^21^, ^22^, ^23^ as well as their high electrical conductivity and resistance to corrosion.^24^ Also, carbides have been utilized as supporting catalysts to reduce the employment of novel metals,^24^, ^25^ Therefore, it is expected that Pt decoration on molybdenum carbide is a cost effective and efficient strategy for facilitating the electrocatalytic HER performance.

To utilize Pt in this method, it is important to fabricate the nanoarchitecture of molybdenum carbides. Recently, there have been addressed various researches about nanoarchitectured molybdenum carbides coupled with conducting carbonaceous materials to increase the effective active sites and accelerate the electrocatalytic kinetics of molybdenum carbides.^1^, ^11^, ^26^, ^27^, ^28^, ^29^, ^30^, ^31^ However, synthesis of molybdenum carbides requires high temperatures over 700 °C in an inert or reductive atmosphere, which leads to the coalescence of the nanoarchitecture and results in the reduction of the electrochemically active surface area (ECSA).^1^, ^26^, ^27^, ^28^, ^29^, ^30^, ^31^ Thus, there still remain challenges in the preparation of highly active molybdenum carbides nanoarchitectures that have a large amount of catalytic active sites.

In this study, we developed α‐phase MoC_1−_ *_x_* nanoparticles (NPs) decorated with Pt‐NPs (MoC_1−_ *_x_*/Pt‐NPs) as active and stable HER electrocatalysts in acidic and alkaline media. First, ultrafine MoC_1−_ *_x_*‐NPs were synthesized through an electrical wire‐explosion (EWE) process at room temperature using commercial Mo wire. As‐prepared MoC_1−_ *_x_*‐NPs exhibit good electrocatalytic HER activity in both media. Then, a small amount of Pt was decorated onto the MoC_1−_ *_x_*‐NPs through the ethanol oxidation method and subsequent thermal treatment. The MoC_1−_ *_x_*/Pt‐NPs showed a maximization of catalytic activity and exhibited superior performance compared to that of the state‐of‐the‐art Mo*_x_*C‐based HER electrocatalyst as well as stability and durability. The experimental procedures related to material synthesis, characterization, and electrochemical measurements are described in the Supporting Information.

**Figure** **1** schematically illustrates the synthesis procedure of MoC_1−_ *_x_*‐NPs. The electrically superheated Mo wire underwent repeated evaporation by explosion, scattering, and condensation to form MoC_1−_ *_x_*‐NPs that were dispersed in the organic solvent medium (Figure 1a). Oleic acid (C_18_H_34_O_2_) was utilized as a solvent medium because it possesses a high amount of carbon (Figure S1, Supporting Information). After filtering and washing the MoC_1−_ *_x_*‐NPs (Figure S2, Supporting Information), the powder was thermally treated in a reductive atmosphere, with H_2_(5%)/Ar gas flowing (the product is denoted as MoC_1−_ *_x_*‐550‐NPs and MoC_1−_ *_x_*‐600‐NPs after a thermal treatment at 550 and 600 °C, respectively) to improve the HER electrocatalytic activity (Figure 1b). Additional information regarding the synthesis of MoC_1−_ *_x_*‐NPs through the EWE process is described in Note S1 and Figures S1 and S2 in the Supporting Information.

**Figure 1:**
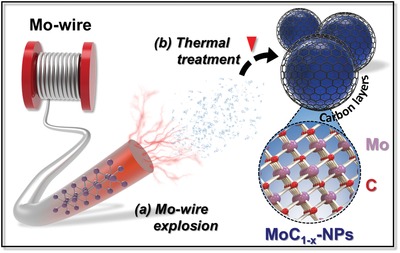
Schematic illustration for the preparation of MoC_1−_ *_x_*‐NPs. a) Mo‐wire explosion and b) thermal treatment process.

**Figure** **2**a shows the X‐ray diffraction (XRD) patterns of MoC_1−_ *_x_*‐EWE‐NPs and their thermally treated samples at 550 and 600 °C. For the MoC_1−_ *_x_*‐EWE‐NPs (lower graph in Figure 2a), all reflected peaks in the pattern match that of the cubic structure α‐phase MoC_1−_ *_x_* (α‐MoC_1−_ *_x_*; Joint Committee on Powder Diffraction Standards (JCPDS) No. 89‐2868). According to the Mo‐C phase diagram, α‐MoC_1−_ *_x_* is a metastable and high‐temperature phase which can be formed at above 2000 °C and below its melting point.^32^ It is expected that evaporated Mo reacts with the carbon at high temperatures, simultaneously undergoing condensation and quenching in the oleic acid, which produces α‐MoC_1−_ *_x_*. The grain size of MoC_1−_ *_x_*‐EWE‐NPs is calculated to be 18.2 nm through the Debye–Scherrer equation, which is consistent with the results of the following transmission electron microscopy (TEM) images.

**Figure 2:**
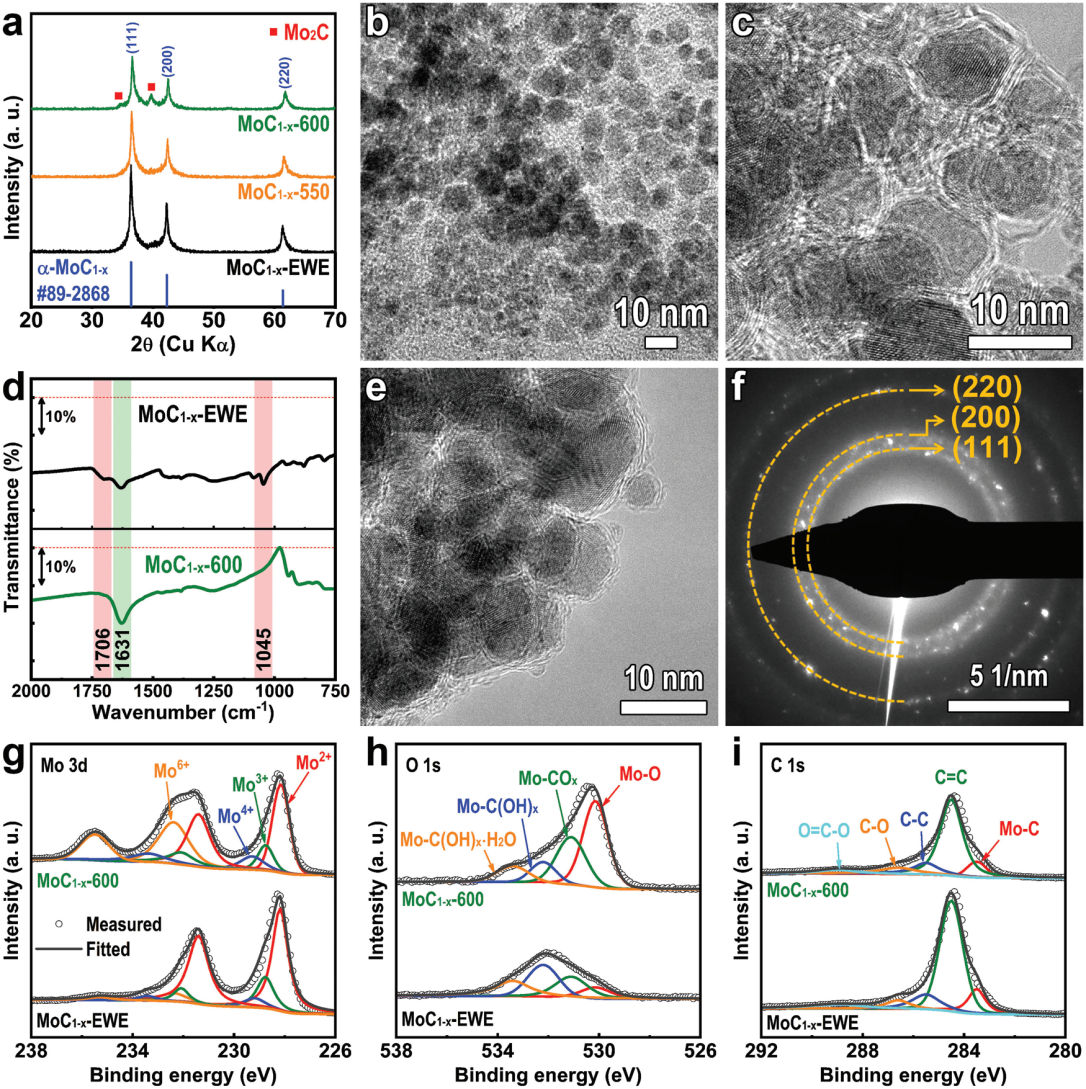
Preparation of ultrafine, MoC_1−_ *_x_*‐NPs. a) XRD patterns of MoC_1−_ *_x_*‐EWE‐NPs, MoC_1−_ *_x_*‐550‐NPs and MoC_1−_ *_x_*‐600‐NPs. b,c) TEM images of MoC_1−_ *_x_*‐EWE‐NPs. d) FT‐IR curves of MoC_1−_ *_x_*‐EWE‐NPs and MoC_1−_ *_x_*‐600‐NPs. e,f) TEM image and SAED patterns of MoC_1−_ *_x_*‐600‐NPs. g–i) Mo 3d, O 1s, and C 1s XPS spectra of MoC_1−_ *_x_*‐EWE‐NPs and MoC_1−_ *_x_*‐600‐NPs.

Figure 2b and Figure S3 in the Supporting Information show the TEM and scanning electron microscopy (SEM) images of MoC_1−_ *_x_*‐EWE‐NPs. Most of the MoC_1−_ *_x_*‐EWE‐NPs are ultrafine, nanosized particles with a diameter of below 20 nm. In the high‐resolution TEM (HRTEM) image, well‐defined nanocrystals that possess continuous lattice fringes are observed (Figure 2c). In addition, MoC_1−_ *_x_*‐NPs are encapsulated in a few carbon layers, which imply that the carbon in oleic acid is transformed into carbon layers during the explosion. Figure 2d shows the Fourier transform infrared (FT‐IR) curves of MoC_1−_ *_x_*‐EWE‐NPs and MoC_1−_ *_x_*‐600‐NPs. In MoC_1−_ *_x_*‐EWE‐NPs, two peaks at 1045 and 1706 cm^−1^ indicate that C—O and C=O functional groups are attached.^33^, ^34^ These peaks are not observed in the curves of MoC_1−_ *_x_*‐600‐NPs, indicating the removal of unnecessary organic compounds. In elemental analysis, it is found that the carbon content is reduced from 28.4 to 17.4 wt% after thermal treatment (Table S1, Supporting Information). However, a sharp peak at 1631 cm^−1^ corresponding to C=C is observed after thermal treatment, which implies the existence of carbon layers. The α‐MoC_1−_ *_x_* phase is not altered after thermal treatment at 550 °C (middle graph in Figure 2a). Although the partial β‐Mo_2_C phase was formed at 600 °C, the α‐MoC_1−_ *_x_* phase is mainly detected in the XRD pattern of MoC_1−_ *_x_*‐600‐NPs (upper graph of Figure 2a). Further TEM analysis of MoC_1−_ *_x_*‐600‐NPs confirms the microstructural information of MoC_1−_ *_x_*‐600‐NPs. Figure 2e shows that no significant change occurs in MoC_1−_ *_x_*‐600‐NPs as they maintain their initial particle size and morphology as well as carbon layers on the surface. Furthermore, selected area electron diffraction (SAED) patterns reveal the formation of α‐MoC_1−_ *_x_* (Figure 2f). Therefore, high‐purity MoC_1−_ *_x_*‐NPs, encapsulated in a few graphitic carbon layers, can be obtained through EWE, followed by a thermal treatment process.

The chemical states of MoC_1−_ *_x_*‐EWE‐NPs and MoC_1−_ *_x_*‐600‐NPs were characterized through X‐ray photoelectron spectroscopy (XPS) analysis. In the Mo 3d XPS spectra (Figure 2g), Mo has four valence states (2+, 3+, 4+, and 6+) in both NPs. The higher valence states of the Mo^4+^/Mo^6+^ peaks at 229.3/232.3 eV (Mo 2d_5/2_) and 233.5/235.5 eV (Mo 3d_3/2_) are attributed to the partial oxidized bonding on the surface of MoC_1−_ *_x_*, resulting from exposure to air,^35^, ^36^ while the lower valence states of Mo^2+^/Mo^3+^ peaks at 228.2/228.8 eV (Mo 3d_5/2_) and 231.4/232.1 eV (Mo 3d_3/2_) are consistent with the Mo—C bonding in MoC_1−_ *_x_*.^31^, ^35^, ^36^ There are no significant changes in Mo^2+^ and Mo^3+^ peaks after thermal treatment, which also confirms the maintenance of the α‐MoC_1−_ *_x_* phase. The increase of Mo^4+^ and Mo^6+^ oxidation peaks after thermal treatment is possibly attributed to greater exposure of the Mo—C surface to air, resulting from the removal of organic compounds during thermal treatment. Indeed, shown in O 1*s* XPS spectra (Figure 2h), certain hydroxide and carbonate peaks are dominant in that of MoC_1−_ *_x_*‐EWE‐NPs, while these peaks are decreased and Mo—O peaks are increased in the spectra of MoC_1−_ *_x_*‐600‐NPs.^37^ The C 1*s* XPS spectra show the Mo—C peak at 283.5 eV and C—C peaks at 284.5 eV (Figure 2i), which are indexed in both NPs, which confirm the formation of MoC_1−_ *_x_* and C layers.^37^

The electrocatalytic HER activities of MoC_1−_ *_x_*‐NPs were measured in acidic (0.5 m H_2_SO_4_) and alkaline (1 m KOH) media in a three‐electrode cell configuration (mass loading: 2 mg cm^−2^). **Figure** **3**a,b presents the polarization curves of MoC_1−_ *_x_*‐NPs in acidic medium and the Tafel plots derived from the polarization curves. In spite of their possession of ultrafine particles, MoC_1−_ *_x_*‐EWE‐NPs demonstrate inferior catalytic activity. However, the overpotential is significantly decreased from 345 to 180 mV at 10 mA cm^−2^ after thermal treatment at 600 °C. There is also an increase of current density with a slight increase in overpotential. In addition, Tafel slopes also decrease from 124 to 59 mV dec^−1^, which indicates higher reaction kinetics after thermal treatment.

**Figure 3:**
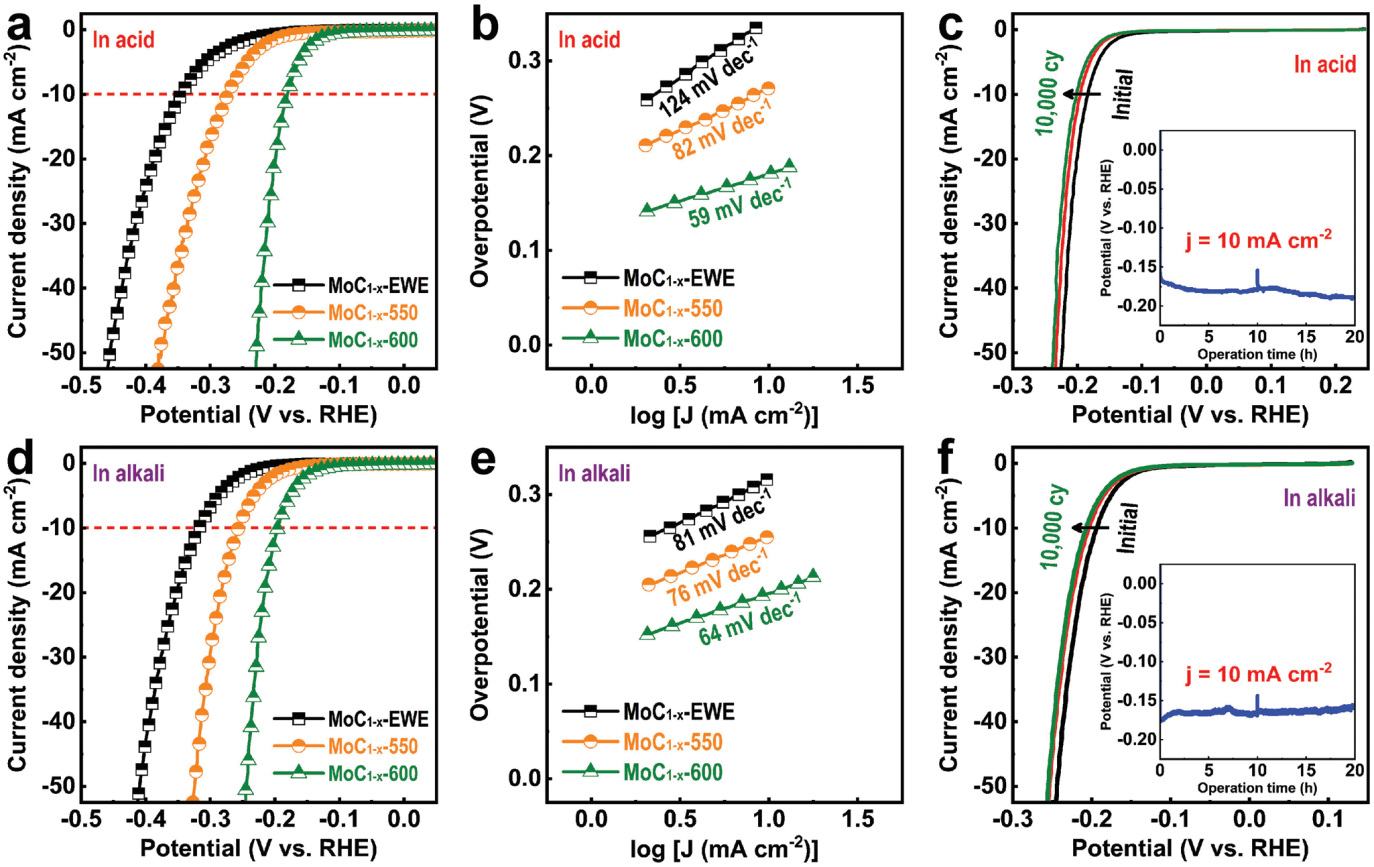
The electrocatalytic HER performance of MoC_1−_ *_x_*‐NPs a–c) in acidic and d–f) alkaline media. a,d) Polarization curves of MoC_1−_ *_x_*‐EWE‐NPs, MoC_1−_ *_x_*‐550‐NPs, and MoC_1−_ *_x_*‐600‐NPs at a scan rate of 5 mV s^−1^ and b,e) the corresponding Tafel plots. c,f) Polarization curves of MoC_1−_ *_x_*‐600‐NPs after the 1st, 5000th, and 10 000th CV and the chronopotentiometric test at a constant current density of 10 mA cm^−2^ (inset).

The HER mechanism generally follows a two‐step reaction. The first step is the Volmer reaction, in which the protons (H^+^) in the electrolyte are trapped by the electrons on the surface of the electrocatalyst, forming the intermediate adsorbed H (H_ads_). Next, the second step is the Heyrovsky or Tafel reaction, in which electrochemical or chemical desorption occurs in catalysts. The thermodynamic Tafel slopes of the aforementioned Volmer and Heyrovsky/Tafel reactions are calculated to be 120, 40, and 30 mV dec^−1^ at room temperature, respectively.^38^ For MoC_1−_ *_x_*‐EWE‐NPs, a Tafel slope of 124 mV dec^−1^ indicates that electrochemical H^+^ adsorption is the rate‐limiting step, which is because of the presence of impurities on the surface of the NPs.^39^, ^40^ However, Tafel slope of 59 mV dec^−1^ in MoC_1−_ *_x_*‐600‐NPs (which is close to 40 mV dec^−1^) implies the improvement of electrochemical H^+^ adsorption. Thus, electrochemical desorption is the rate‐limiting step during H_2_ production on MoC_1−_ *_x_*‐600‐NPs.

The double‐layer capacitances (*C*_dl_) of MoC_1−_ *_x_*‐NPs demonstrate an increase in number of electrochemical active sites.^41^ *C*_dl_ was measured by cyclic voltammetry (CV) at scan rates from 5 to 200 mV s^−1^ in nonfaradic potentials from 0.1 to 0.3 V (vs reversible hydrogen electrode) (Figure S4, Supporting Information). From the calculations, MoC_1−_ *_x_*‐EWE‐NPs had a low *C*_dl_ of 3 mF cm^−2^, while the *C*_dl_ of MoC_1−_ *_x_*‐550‐NPs and MoC_1−_ *_x_*‐600‐NPs are significantly increased at 18 and 27 mF cm^−2^, respectively. This confirms that the removal of impurities through thermal treatment leads to an increase in ECSA. Electrochemical impedance spectroscopy analysis of the MoC_1−_ *_x_*‐NPs was performed in an acidic environment. Figure S5 in the Supporting Information shows the Nyquist plots that were measured at an overpotential of 180 mV including their fitted lines determined by an equivalent circuit, and their fitting data were summarized in Table S2 in the Supporting Information. From the fitting, all plots consist of two semicircles. The semicircle at high frequencies corresponds to the surface porosity of the electrode (*R*_sf_), while the other semicircle, at low frequencies, is attributed to the kinetics of the electrocatalysts that are related to charge transfer resistance (*R*_ct_).^23^ On the basis of the fitting data, MoC_1−_ *_x_*‐600‐NPs had lower *R*_sf_ (0.5 Ω) than MoC_1−_ *_x_*‐EWE‐NPs (25 Ω). This implies that MoC_1−_ *_x_*‐600‐NPs would exhibit higher surface porosity and enable rapid diffusion of ions and electrons compared to MoC_1−_ *_x_*‐EWE‐NPs, due to the removal of impurities on the surface of the MoC_1−_ *_x_*‐NPs after thermal treatment. This result can support the tendency of *C*_dl_ increase after thermal treatment. Also, there is a stronger relationship between *R*_ct_ and the electrocatalytic performance of MoC_1−_ *_x_*‐NPs that MoC_1−_ *_x_*‐600‐NPs showed lower *R*_ct_ (10 Ω) than MoC_1−_ *_x_*‐EWE‐NPs (480 Ω). Because of the removal of impurities, as well as greater graphitization of C layers (Figure S6, Supporting Information), the electrocatalytic kinetics of MoC_1−_ *_x_*‐600‐NPs are significantly enhanced.

The long‐term stability and durability of MoC_1−_ *_x_*‐600‐NPs were also measured. Figure 3c shows the polarization curves of MoC_1−_ *_x_*‐600‐NPs after the continuous CV, cycled from 0 to −200 mV at a scan rate of 100 mV s^−1^ in an acidic medium. Compared with the initial state, slight shifts in the polarization curves (15 mV at 10 mA cm^−2^) are observed after the 10 000th cycle, which indicates the high stability of MoC_1−_ *_x_*‐NPs after the CV. In addition, MoC_1−_ *_x_*‐600‐NPs demonstrate high durability in the chronopotentiometry test, as measured at a constant current density of 10 mA cm^−2^ (inset of Figure 3c). Compared with the initial potential, it shows an increase of overpotential (20 mV) after 20 h, with continuous H_2_ production. Thus, the stability and durability of MoC_1−_ *_x_*‐NPs are ascertained by continuous cycling and chronopotentiometric tests in an acidic medium.

The electrocatalytic performance of MoC_1−_ *_x_*‐NPs in an alkaline medium was also measured. In the polarization curves and Tafel plots (Figure 3d,e), the electrocatalytic activity of MoC_1−_ *_x_*‐NPs improves after thermal treatment, as they do in an acidic medium. Interestingly, MoC_1−_ *_x_*‐600‐NPs exhibit an overpotential and Tafel slope of 195 mV and 64 mV dec^−1^ at 10 mA cm^−2^ in an alkaline medium, which is similar to that measured in an acidic medium. In general, it has been known that the electrocatalytic HER activities of electrocatalysts in an alkaline medium are two to three orders of magnitude lower than in an acidic medium, owing to the inefficient dissociation of water in proton adsorption in an alkaline environment.^42^, ^43^, ^44^ However, in a basic environment, MoC_1−_ *_x_*‐600‐NPs demonstrate an electrocatalytic activity similar to that measured in an acidic environment. This suggests that MoC_1−_ *_x_*‐NPs exhibit an active water dissociation process in a basic environment. Additionally, the stability and durability of MoC_1−_ *_x_*‐600‐NPs are confirmed in this medium (Figure 3f). Meanwhile, β‐Mo_2_C phase were more increased as MoC_1−_ *_x_*‐EWE‐NPs were thermally treated above 600 °C. However, there was no significant difference in HER performance of all samples in both acidic and alkaline media (Figure S7, Supporting Information). Although it is known that β‐Mo_2_C phase exhibits the higher HER performance than α‐MoC_1−_ *_x_* phase, α‐MoC_1−_ *_x_*‐NPs can also generate a highly active HER electrocatalytic performance.

Through utilization of MoC_1−_ *_x_*‐NPs, MoC_1−_ *_x_*/Pt‐NPs containing a small amount of Pt were synthesized and evaluated for their electrocatalytic HER ability. As shown in **Figure** **4**a, Pt was decorated on the surface of MoC_1−_ *_x_*‐NPs through the ethanol oxidation method,^45^ and then the MoC_1−_ *_x_*/Pt‐NPs were thermally treated at 600 °C in a reductive environment. The inductively coupled plasma atomic emission spectroscopy and energy‐dispersive spectroscopy (EDS) mapping with SEM estimate that the Pt content in the MoC_1−_ *_x_*/Pt‐600‐NPs is 2.7–3 wt% (Table S3, Figure S8, and Note S2, Supporting Information). Figure 4b shows the XRD patterns of MoC_1−_ *_x_*/Pt and MoC_1−_ *_x_*/Pt‐600 NPs. After incorporation with Pt, no phase change occurred in MoC_1−_ *_x_*/Pt‐NPs compared to that of MoC_1−_ *_x_*‐EWE‐NPs. Likewise, MoC_1−_ *_x_*/Pt‐600‐NPs exhibit the same α‐MoC_1−_ *_x_* phase, indicating that Pt has little influence. No characterization peaks of Pt are observed in the XRD patterns, possibly as a result of the minor amount of Pt in MoC_1−_ *_x_*/Pt‐NPs.

**Figure 4:**
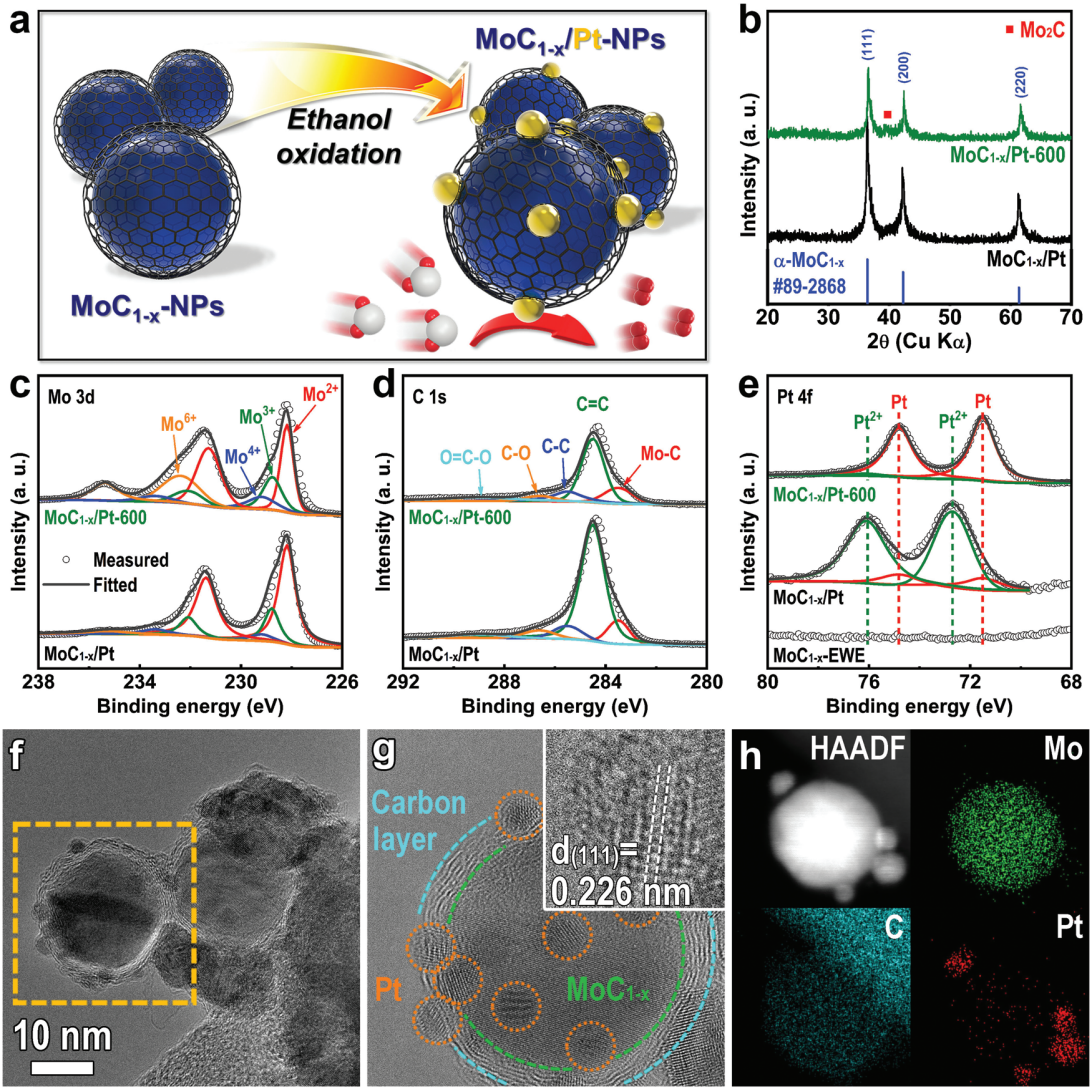
Pt decoration onto MoC_1−_ *_x_*‐NPs. a) Schematic illustration of MoC_1−_ *_x_*/Pt‐NPs. b) XRD patterns of MoC_1−_ *_x_*/Pt‐NPs and MoC_1−_ *_x_*/Pt‐600‐NPs. c–e) Mo 3d, C 1s, and Pt 4f XPS spectra of MoC_1−_ *_x_*/Pt‐NPs and MoC_1−_ *_x_*/Pt‐600‐NPs. f,g) TEM images and h) EDS elemental mapping of MoC_1−_ *_x_*/Pt‐600‐NPs.

The chemical states of MoC_1−_ *_x_*/Pt‐NPs and MoC_1−_ *_x_*/Pt‐600‐NPs were characterized by XPS analysis. As shown in Figure 4c and d, both NPs exhibit the same Mo and C valence states in Mo 3d and C 1s XPS spectra compared to those of MoC_1−_ *_x_*‐EWE‐NPs and MoC_1−_ *_x_*‐600‐NPs. This indicates that there is no change of chemical states during the Pt coating and the subsequent thermal treatment. Figure 4e shows the Pt 4f XPS spectra of MoC_1−_ *_x_*/Pt‐NPs and MoC_1−_ *_x_*/Pt‐600‐NPs. Pt 4f XPS spectra are observed around 70–80 eV, which confirms the existence of Pt on MoC_1−_ *_x_*‐NPs. The Pt 4f spectrum of MoC_1−_ *_x_*/Pt‐NPs is deconvoluted to high‐intensity peaks at 72.7/76.1 eV and low‐intensity peaks at 71.5/74.8 eV, which correspond to Pt^2+^ and metallic Pt, respectively.^46^ After thermal treatment, almost all of the Pt^2+^ were converted into metallic Pt because of the thermal treatment in a reductive atmosphere.

The morphology of MoC_1−_ *_x_*/Pt‐600‐NPs was investigated by TEM analysis. After Pt incorporation and thermal treatment, NPs with diameters of 2–3 nm were distributed on the larger MoC_1−_ *_x_* NP that was encapsulated in carbon layers, without aggregation (Figure 4f,g). The HRTEM image of these smaller NPs (inset of Figure 4g) shows that the lattice spacing of 0.226 nm is indexed to the (111) plane of the cubic structure of Pt (JCPDS No. 04‐0802). The high‐angle annular dark field scanning TEM EDS elemental mapping also demonstrates the formation of Pt‐NPs on the surface of MoC_1−_ *_x_*‐NPs (Figure 4h). During the Pt‐decoration process (during either ethanol oxidation or thermal treatment), partial carbon on the surface of α‐MoC_1−_ *_x_*‐NPs would be oxidized to CO*_x_* simultaneously with reduction of Pt^2+^ to Pt^0^, resulting in the formation of Pt‐NPs on the carbon layer.

The electrocatalytic HER performance of MoC_1−_ *_x_*/Pt‐600‐NPs was investigated, along with the performance of the benchmark Pt(20%)/C catalyst for comparison. **Figure** **5**a,b presents the polarization curves of MoC_1−_ *_x_*/Pt‐600‐NPs in media and their Tafel plots. Impressively, MoC_1−_ *_x_*/Pt‐600‐NPs require only a 30 mV overpotential to achieve a current density of 10 mA cm^−2^ in acidic medium, and their Tafel slope is estimated to 31 mV dec^−1^, which is comparable to that of commercial Pt/C. Because of the uniform distribution and effective incorporation of Pt‐NPs on to MoC_1−_ *_x_*‐NPs, a small amount of Pt can maximize the electrocatalytic HER activity in an acidic environment. The MoC_1−_ *_x_*/Pt‐600‐NPs also exhibit an excellent electrocatalytic activity with a small overpotential of 67 mV at 10 mA cm^−2^ and a Tafel slope of 55 mV dec^−1^ in an alkaline medium. This is also comparable to that of commercial Pt/C. Note that the high HER performance of MoC_1−_ *_x_*/Pt‐NPs is superior to that of state‐of‐the‐art Mo*_x_*C‐based HER electrocatalysts in acidic and alkaline media (Table S4, Supporting Information). To compare the intrinsic electrocatalytic HER activity of Pt/C and MoC_1−_ *_x_*/Pt‐600‐NPs, the *C*_dl_ of Pt/C and MoC_1−_ *_x_*/Pt‐600‐NPs were measured and the current density (right graph in Figure 5a) was normalized to the ECSA (Figure S9 and Note S3, Supporting Information). As shown in Figure 5c, a lower overpotential is required to achieve the same current density in MoC_1−_ *_x_*/Pt‐600‐NPs compared to that of Pt/C. This tendency is more pronounced at a higher current density, which indicates that MoC_1−_ *_x_*/Pt‐600‐NPs have a higher intrinsic catalytic activity than Pt/C. As aforementioned in Figure 3, the facile water dissociation property of MoC_1−_ *_x_*‐NPs provides more active sites for the formation of intermediate H_ads_ in an alkaline medium. Therefore, the combination of MoC_1−_ *_x_* and Pt increases the electrocatalytic kinetics of Pt catalysis for HER in an alkaline medium.

**Figure 5:**
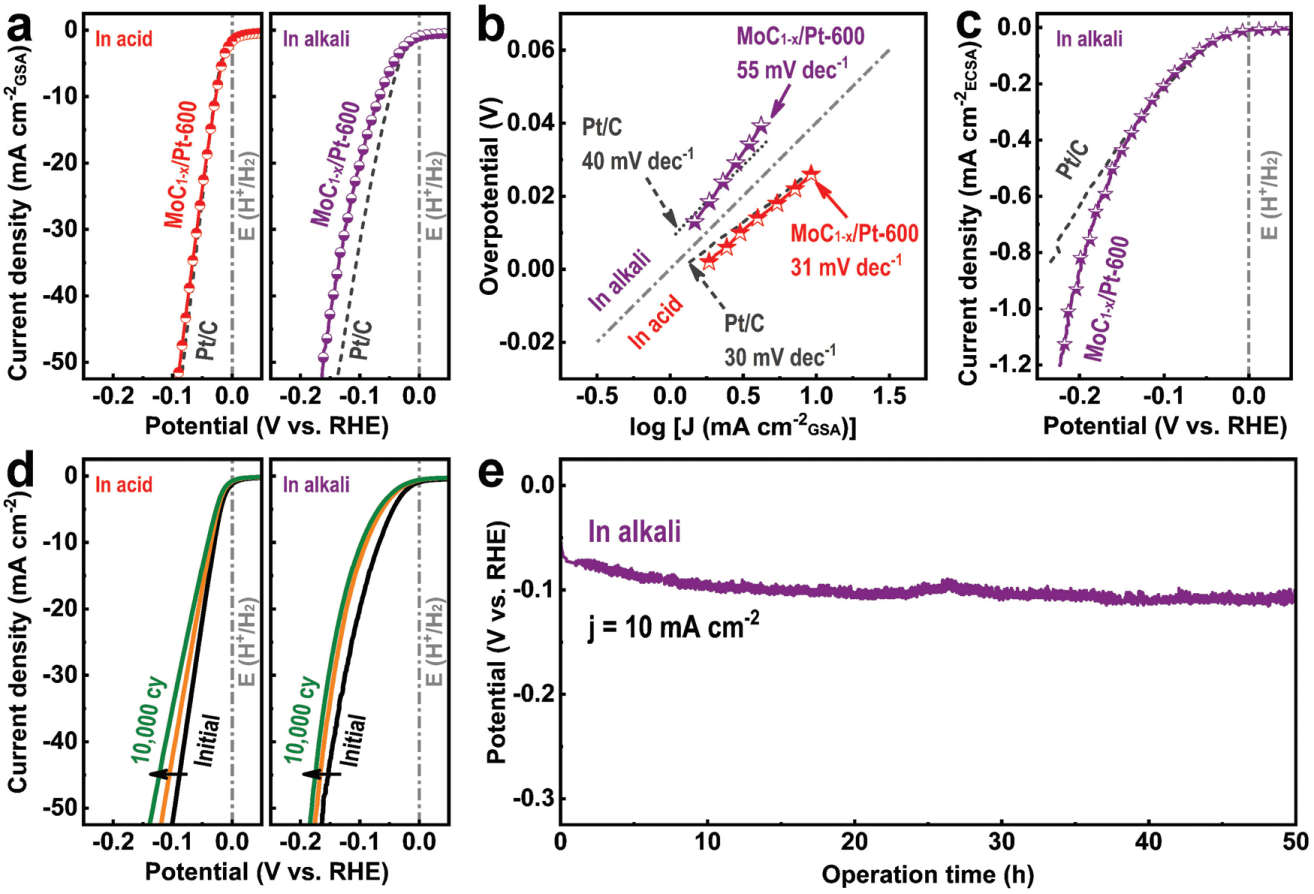
Electrocatalytic HER performance of MoC_1−_ *_x_*/Pt‐600‐NPs and Pt/C in acidic and alkaline media. a) Polarization curves at a scan rate of 5 mV s^−1^ and b) corresponding Tafel plots. c) Polarization curves with current densities normalized to the ECSA. d) Polarization curves after the 1st, 5000th, and 10 000th CV. e) Chronopotentiometric curve at a constant current density of 10 mA cm^−2^ in alkaline medium.

Figure 5d shows the polarization curves of MoC_1−_ *_x_*/Pt‐600‐NPs after continuous CV, cycled from 0 to −50 mV and from 0 to −100 mV at a scan rate of 100 mV s^−1^ in acidic and alkaline media, respectively. Compared with the initial state, slight shifts of the polarization curves (10 and 20 mV at 10 mA cm^−2^ in acidic and alkaline media, respectively) are observed after the 10 000th cycle, which indicates that MoC_1−_ *_x_*/Pt‐600‐NPs have high stability during continuous CV. This stability originates from that of MoC_1−_ *_x_*‐600‐NPs as well as the effective incorporation of Pt with MoC_1−_ *_x_*‐NPs. Additionally, MoC_1−_ *_x_*/Pt‐600‐NPs show high durability during the chronopotentiometry test, as measured at a constant current density of 10 mA cm^−2^ (Figure 5e). Compared with the initial potential, it shows only a slight degradation of 25 mV after 50 h in alkaline medium, respectively, with continuous H_2_ production. Although surface chemical states of MoC_1−_ *_x_*/Pt‐600‐NPs were changed, there was no significant change in XRD patterns of MoC_1−_ *_x_*/Pt‐600‐NPs after catalytic test in acidic and alkaline media (Figure S10, Supporting Information), indicating high microstructural stability of MoC_1−_ *_x_*/Pt‐600‐NPs during the catalytic test. In addition, the Faradaic efficiency of MoC_1−_ *_x_*/Pt‐600‐NPs is measured to be ≈100% for the HER in alkaline medium (Figure S11, Supporting Information). Thus, the high stability and durability of MoC_1−_ *_x_*/Pt‐600‐NPs are ascertained.

In summary, we have developed novel MoC_1−_ *_x_*/Pt‐NPs electrocatalysts for efficient H_2_ production in acidic and alkaline media, where Pt‐NPs, making up 2.7–3 wt%, were decorated onto the MoC_1−_ *_x_*‐NPs, which were encapsulated in carbon layers. The ultrafine, MoC_1−_ *_x_*‐NPs were synthesized through an electrical Mo‐wire explosion in oleic acid and thermal treatment. MoC_1−_ *_x_*‐NPs exhibit good electrocatalytic activity for HER in media of both ends of the pH scale. They demonstrate fast reaction kinetics in an alkaline medium compared to an acidic medium. Pt‐NPs with diameters of 2–3 nm were incorporated into MoC_1−_ *_x_*‐NPs. MoC_1−_ *_x_*/Pt‐NPs exhibit an electrocatalytic activity comparable to that of commercial Pt/C and even demonstrate a higher intrinsic catalytic activity in an alkaline medium. In addition, high stability and durability are confirmed in both media. Therefore, our material design suggests that MoC_1−_ *_x_*‐NPs operate as effective catalysts and support the minimal utilization of Pt for efficient H_2_ production.

## Conflict of Interest

The authors declare no conflict of interest.

## Supporting information

Supplementary
