# Supplementary material for: Ultrafine α‐Phase Molybdenum Carbide Decorated with Platinum Nanoparticles for Efficient Hydrogen Production in Acidic and Alkaline Media
Source: Adv Sci (Weinh). 2019 Feb 21;6(8):1802135. doi: 10.1002/advs.201802135 (PMC6468960; doi:10.1002/advs.201802135)
Supplement: Supplementary file 1 — Supplementary [file ADVS-6-1802135-s001.pdf]

Copyright WILEY-VCH Verlag GmbH & Co. KGaA, 69469 Weinheim, Germany, 2019.

ADVANCED SCIENCE
Open Access

Supporting Information

for *Adv. Sci.,* DOI: 10.1002/advs.201802135

Ultrafine α-Phase Molybdenum Carbide Decorated with Platinum Nanoparticles for Efficient Hydrogen Production in Acidic and Alkaline Media

*Hee Jo Song, Myeong-Chang Sung, Hyunseok Yoon, Bobae Ju, and Dong-Wan Kim**

Copyright WILEY-VCH Verlag GmbH & Co. KGaA, 69469 Weinheim, Germany, 2019.

Supporting Information

Ultrafine α-phase molybdenum carbide decorated with platinum nanoparticles for efficient hydrogen production in acidic and alkaline media

Hee Jo Song, Myeong-Chang Sung, Hyunseok Yoon, Bobae Ju, and Dong-Wan Kim*

Experimental Section

Synthesis of MoC_{1-x}-NPs: Ultrafine MoC_{1-x}-NPs were prepared through an EWE and a thermal reduction process. Commercial Mo wires (diameter = 0.2 mm) were utilized as precursors. Electrical pulse equipment (NTiminiP, Nano Tech, Korea) was employed to fabricate MoC_{1-x}-NPs. EWE was performed at a 40 mm feeding distance and 320 V charging voltage in the media, including methanol, ethanol, isopropyl alcohol (IPA), and oleic acid. After wire-explosion, the MoC_{1-x} was washed and filtered several times with methanol, and then dried in a vacuum oven at 70°C. For the improvement of their electrocatalytic activity, as-exploded MoC_{1-x}-NPs were thermally treated in the tube furnace that was flowing H_2(5%)/Ar gas.

Pt decoration on MoC_{1-x}-NPs: Pt decoration on MoC_{1-x}-NPs is referred to as the aerobic alcohol oxidation process.[S1] As-exploded MoC_{1-x}-NPs (120 mg) were dispersed in absolute ethanol (60 mL) and ultrasonicated for 30 min, and then transferred to a three-neck, round-bottom flask. An absolute ethanol solution (2.54 mL) containing H_2PtCl_6 (52.8 mg, Sigma Aldrich) was added to the flask. After mixing, it was refluxed at 70 °C for 2 h with magnetic stirring. After 2 h, 0.2 M NaOH (1.27 mL) solution was added to the flask and refluxed for an additional 30 min to ensure the reduction of the Pt. The mixture was filtered, washed with absolute ethanol and de-ionized water several times, and dried in an electrical oven. In order to control the MoC_{1-x}/Pt phase, MoC_{1-x}/Pt-NPs were heat-treated in the tube furnace in a reductive atmosphere.

Characterization: XRD patterns of the powder samples were obtained with a Rigaku SmartLab through Cu Ka radiation at a scan rate of 5° min^{-1}. SEM images with EDS mapping were obtained with a Hitachi SU-70. TEM analysis with EDS mapping was conducted with a JEOL JEM-2100F. FT-IR analysis was completed with the Horiba LabRam Aramis IR2.

Chemical analysis was performed by XPS (Theta probe base system, Thermo Fisher Scientific) and Raman spectrometry (LabRam ARAMIS IR2, HORIBA JOBIN YVON). The concentrations of the elements were determined with ICP-AES (OPTIMA 4300DV, PerkinElmer). The elemental analysis was determined with the Vario MICRO cube.

Electrochemical measurements: The HER electrocatalytic measurements were performed in a three-electrode cell configuration that utilized a rotating disk electrode at a rotation speed of 2000 rpm. A glassy carbon electrode (GCE; 5 mm in diameter) was coated with catalyst ink and utilized as the working electrode. The catalyst ink was prepared by dispersing catalyst (20 mg) in water/IPA solution (750/200 μL) and 5 wt% Nafion solution (50 μL), followed by ultrasonication for 30 min. Then, catalyst ink (20 μL) was dropped onto the GCE and dried under an infrared lamp. For comparison, Pt/C (20 wt% Pt, Johnson Matthey) catalytic ink was prepared in the same way. A saturated calomel electrode (SCE) and Hg/HgO electrode were utilized as the reference electrodes in acidic and alkaline media, respectively. A graphite rod was employed as the counter electrode. A nitrogen gas (N_2)-saturated, 0.5 M H_2SO_4, and 1 M KOH solution was the electrolyte. Polarization curves were obtained with linear sweep voltammetry at a scan rate of 5 mV s^{-1}. All potentials were corrected with iR compensation to remove the ohmic potential and converted to the RHE through the following equation: $E_{RHE} = E_{ref} + 0.0591pH + E^0_{ref}$ (where E^0_{SCE} and $E^0_{Hg/HgO}$ are 0.241 and 0.098, respectively). EIS measurements were performed at a frequency that ranged from 100 kHz to 10 mHz. For the chronopotentiometric test, a carbon paper electrode (CPE; 5 mm × 5 mm) that was coated with catalyst ink was employed as a working electrode. The Faradaic efficiency was determined by the downward displacement of water at a current density of 50 mA cm^{-2}.

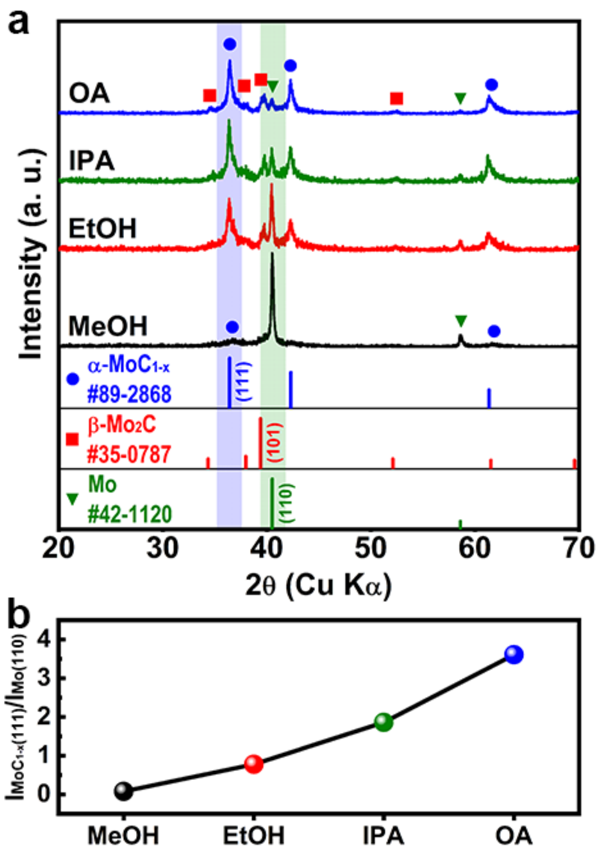

Figure S1. (a) XRD patterns of MoC_{1-x}-NPs exploded in various organic media. (b) The XRD peak intensity ratio of the MoC_{1-x} (111) to the Mo (110) peak.

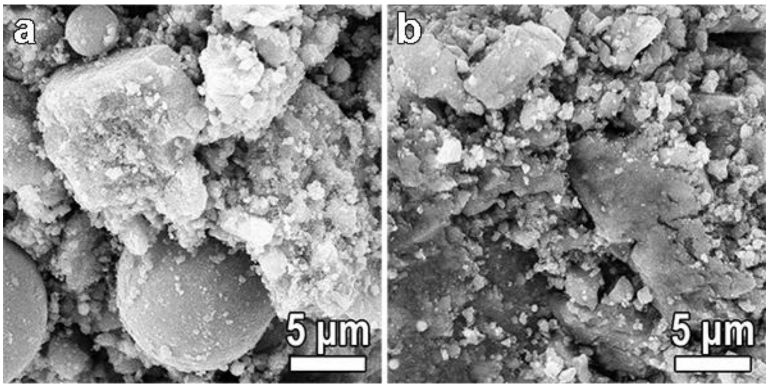

Figure S2. SEM images of (a) MoC_{1-x} before and (b) MoC_{1-x} after filtering with cellulose and micropore filter paper.

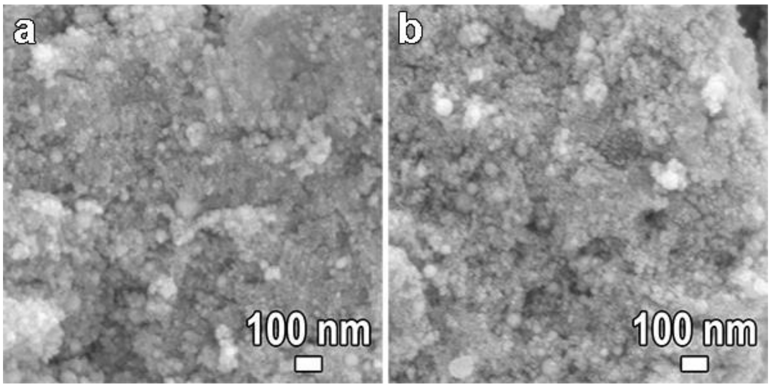

Figure S3. SEM images of MoC_{1-x}-EWE and MoC_{1-x}-600-NPs.

Table S1. Elemental analysis of MoC_{1-x}-NPs and MoC_{1-x}-600-NPs.

Catalyst	Elemental analysis (wt%)			
	C	H	N	S
MoC_{1-x}-EWE	28.4	1.3	0.1	0.2
MoC_{1-x}-600	17.4	0.9	0.1	0.2

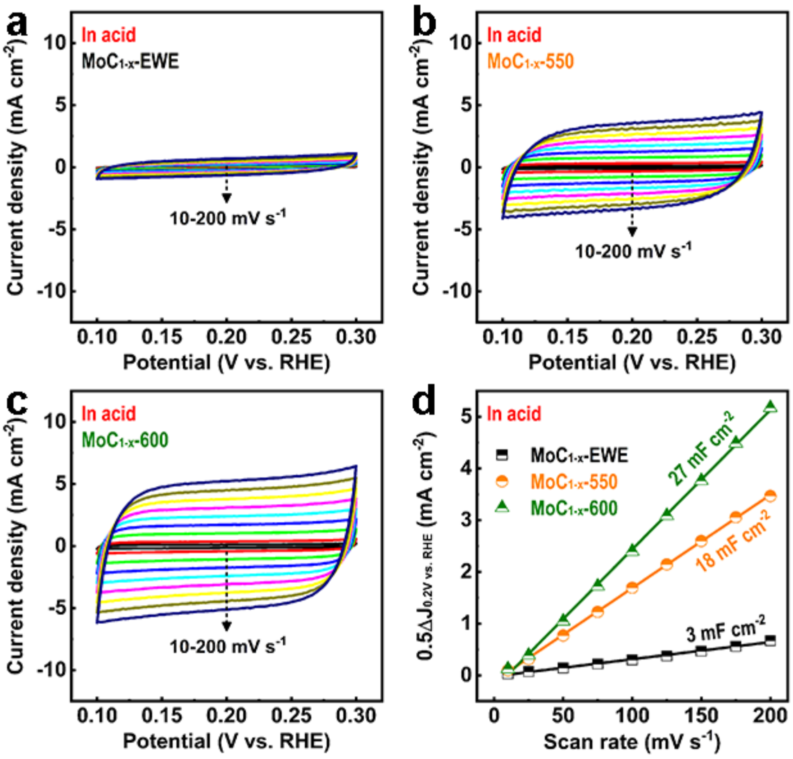

Figure S4. CV curves of (a) MoC_{1-x}-EWE, (b) MoC_{1-x}-550, and (c) MoC_{1-x}-600, cycled between 0.1 and 0.3 V (vs. RHE) at a scan rate from 10 to 200 mV s^{-1} in an acidic medium. (d) Capacitive current densities as a function of scan rate.

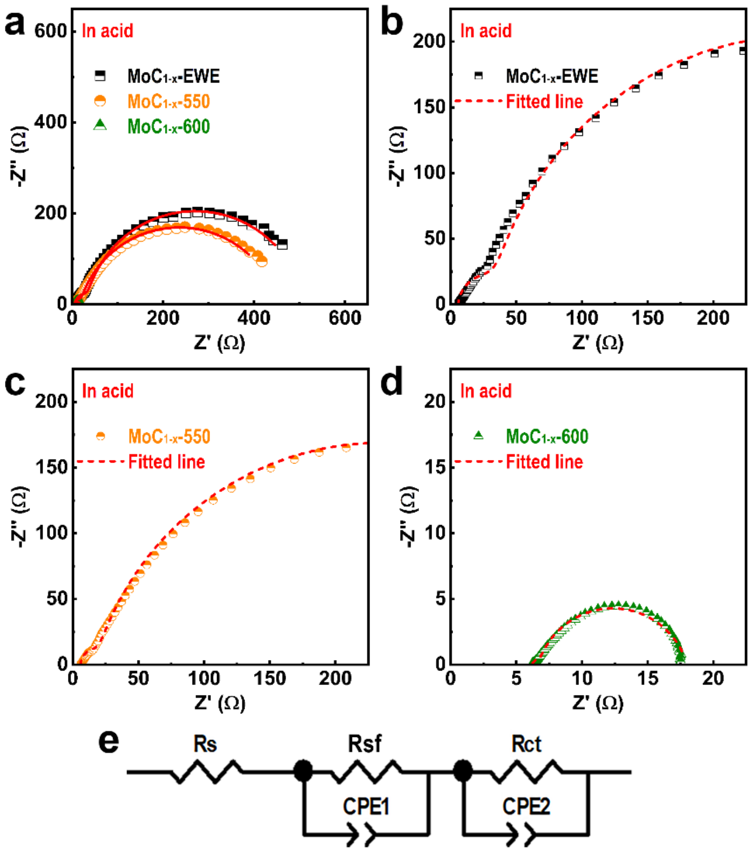

Figure S5. Nyquist plots of MoC_{1-x}-EWE-NPs, MoC_{1-x}-550-NPs, and MoC_{1-x}-600-NPs including their fitted line determined by an equivalent circuit. (a) All three samples, (b) MoC_{1-x}-EWE-NPs, (c) MoC_{1-x}-550-NPs, (d) MoC_{1-x}-600-NPs, (e) equivalent circuit.

Table S2. Fitting data determined by equivalent circuits.

Sample	Element		
	R_s (Ω)	R_{sf} (Ω)	R_{ct} (Ω)
MoC_{1-x}-EWE-NPs	6	25	480
MoC_{1-x}-550-NPs	6.1	11	438
MoC_{1-x}-600-NPs	6	0.5	10

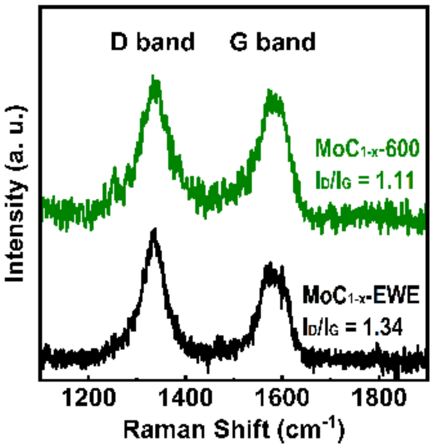

Figure S6. Raman spectra of MoC_{1-x}-EWE-NPs and MoC_{1-x}-600-NPs.

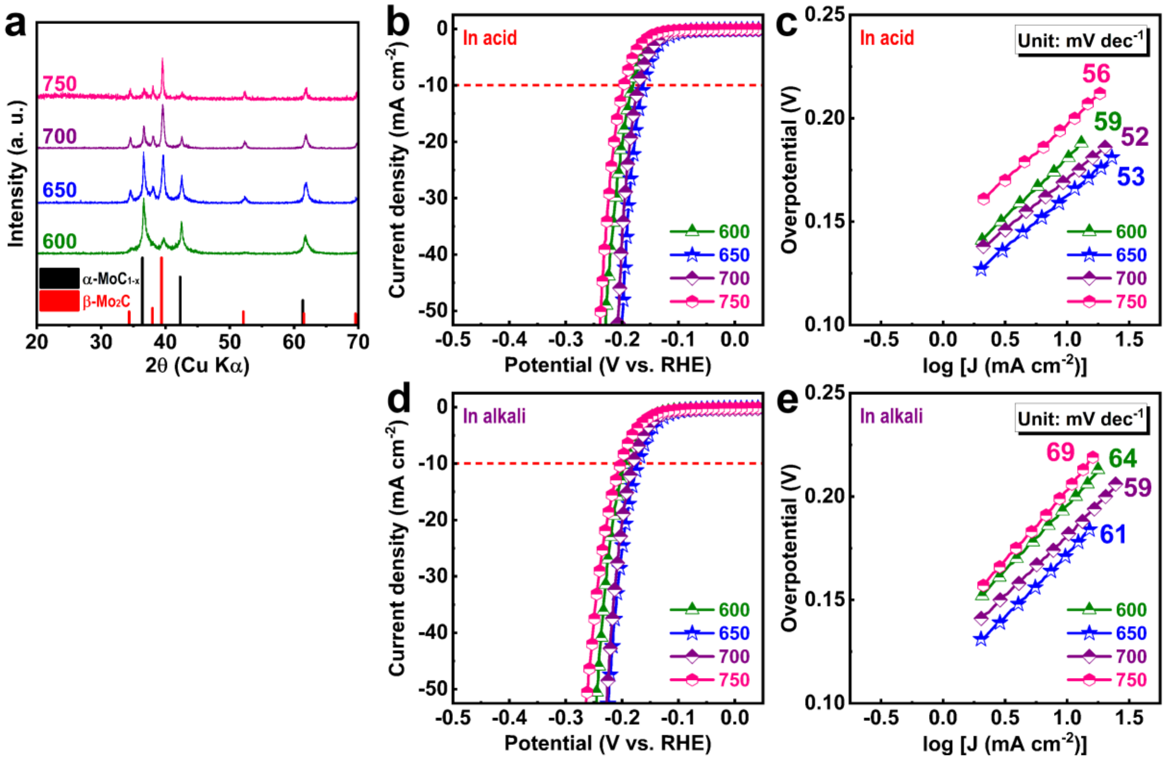

Figure S7. (a) XRD patterns of MoC_{1-x}-600-NPs, MoC_{1-x}-650-NPs, MoC_{1-x}-700-NPs and MoC_{1-x}-750-NPs, and their electrocatalytic performance measured (b,c) in acidic and (d,e) alkaline media. (b,d) Polarization curves at a scan rate of 5 mV s^{-1}, and (c,e) the corresponding Tafel plots.

Table S3. Mo and Pt concentration in MoC_{1-x}/Pt-600-NPs determined by ICP-AES analysis.

	Mo	Pt	MoC
Concentration (ppm)	706729 (measured)	25050 (measured)	795204 (calculated)
Atomic/molecular weight	95.94	195.078	107.9507

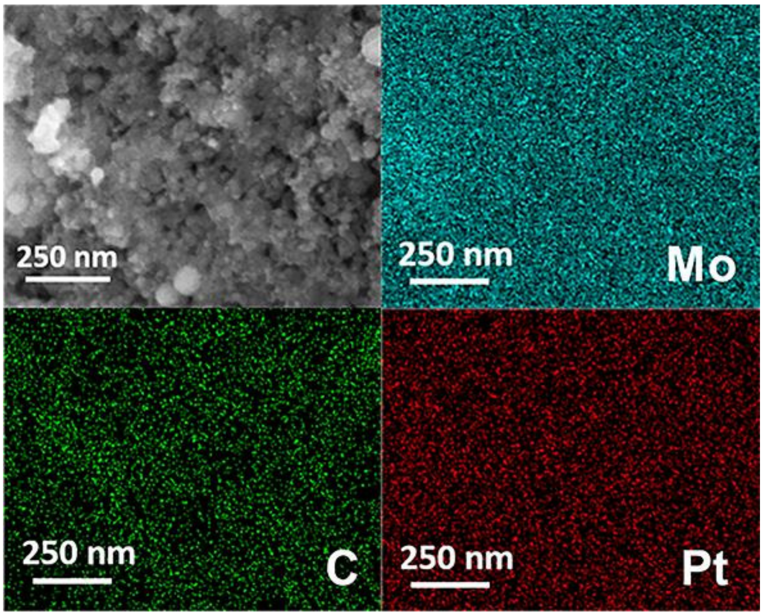

Figure S8. The SEM EDS elemental mapping of MoC_{1-x}/Pt-600-NPs.

Table S4. Comparison of HER performance with α-MoC_{1-x}-based electrocatalysts measured in acidic and alkaline media.

Catalyst	Acid η_{10} (mV)	Acid Tafel slope (mV/dec)	Alkali η_{10} (mV)	Alkali Tafel slope (mV/dec)	Loading (mg/cm^2)	Electrode	Durability (h)	Ref.
α-MoC_{1-x}/Mo_2C NWs	126	43	120	42	0.14	GC	20	S2
H- Induced Mo_2C Hybrid	152	65	121	54	-	GC	5.5	S3
2D porous Mo_2C nanostructures	71	40	92	47	0.55	GC	16	S4
2D Mo_2C	236	73	-	-	-	GC	-	S5
3D hierarchical porous Mo_2C	166	75	139	71	0.4	GC	12	S6
MoC/C NPs	130	63.6	-	-	0.57	GC	20	S7
Mo_2C@ hierarchical porous carbon	145	48.2	-	-	0.357	GC	12	S8
N,P-doped α-MoC_{1-x} NFs	107	65.1	135	57.1	0.265	Carbon fiber	10	S9
Mesoporous Mo_2C@C NW arrays	125	66	-	-	0.2	GC	-	S10
W-doped Mo_xC	125	56	70	44	0.35	GC	24	S11
Mo_2C NPs@	91	-	89	51	1	Ni foam	20	S12

porous carbon								
B-,N-doped Mo_2C NPs@ carbon	-	-	100	62	1	Ni foam	20	S13
Mo/Mo_2C NSs	89	70.7	79	62.8	0.285	GC	20	S14
Mo/α-MoC_{1-x} NRs	110	81.7	-	-	2.1	GC	12	S15
Mo_2C@carbon nanomesh	30	33.7	-	-	0.5	GC	16	S16
Mo_2N-No_2C@ rGO	160	55	150	51	0.337	GC	50	S17
2D Mo_2C–Co	48	39	-	-	1	CP	-	S18
Mo_2C NPs/ graphene/ carbon	70	39	66	37	0.57	GC	20	S19
Mo-Mo_2C	150	55	-	-	0.38	GC	12	S20
Mo_2C/carbon NPs	119	67	85	49	0.35	GC	50	S21
α-MoC_{1-x} nanoparticles	180	59	195	64	2	GC	50	This work
α-MoC_{1-x}/Pt nanoparticles	30	31	67	55	2	GC	50	This work
Pt/C	30	30	50	40	2	GC	-	This work

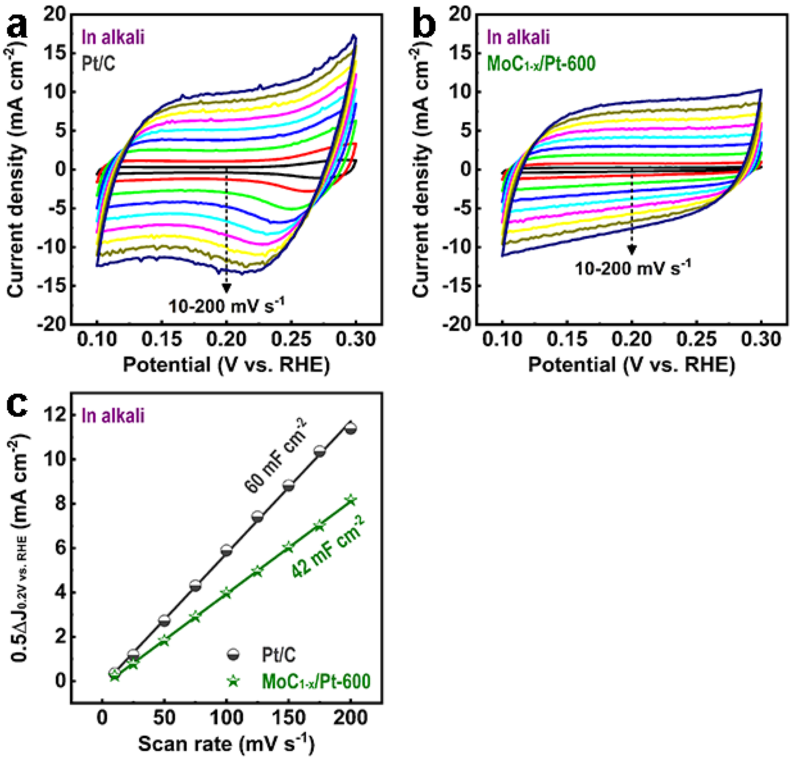

Figure S9. CV curves of (a) Pt/C and (b) MoC_{1-x}/Pt-600-NPs cycled between 0.1 and 0.3 V (vs. RHE) at a scan rate from 10 to 200 mV s^{-1} in an alkaline medium. (c) Capacitive current densities as a function of scan rate.

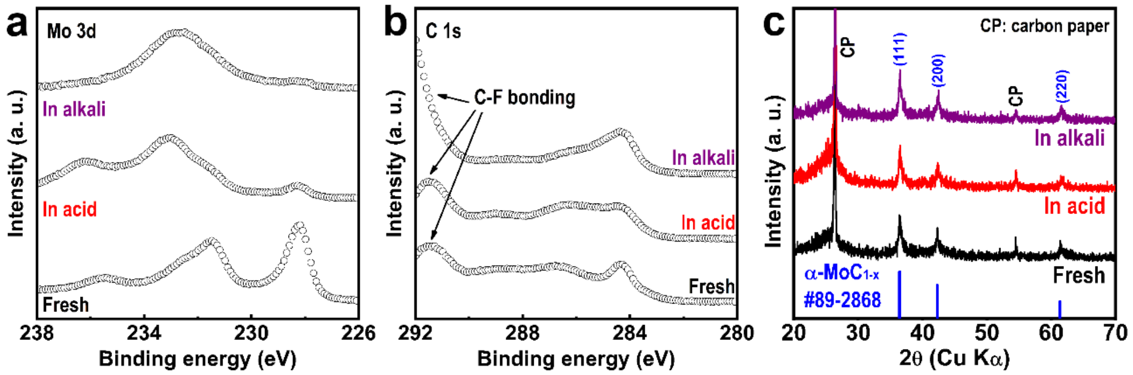

Figure S10. Characterization after electrocatalytic HER test. (a) Mo 2p, (b) C 1s XPS spectra, and (c) XRD patterns of MoC_{1-x}/Pt-600-NPs electrode before and after the chronopotentiometric test in acidic and alkaline media.

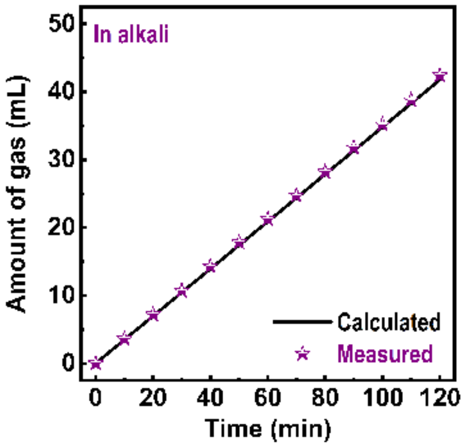

Figure S11. Theoretically calculated and experimentally measured volume of H_2 for the MoC_{1-x}/Pt-600-NPs as a function of time in alkaline medium.

Supplementary Note 1

Synthesis of MoC_{1-x}-EWE-NPs by the electrical Mo-wire explosion process.

Commercial Mo wire was continuously fed with an automatic system. The electrically superheated Mo-wire repeatedly underwent evaporation by explosion, scattering, and condensation, which produced MoC_{1-x}-NPs that were dispersed in a liquid medium. When the Mo wire was exploded in methanol (which has low carbon content), the major product was Mo, as opposed to the MoC_{1-x} phase (Figure S1a). The MoC_{1-x} phase is observed when the Mo wire is exploded in ethanol, IPA, and oleic acid (Figure S1a). Interestingly, as the carbon content of the organic media increases, the XRD peak intensity ratio of MoC_{1-x} (111) to Mo (110) also increases (Figure S1b), which suggests that oleic acid provides more carbon for evaporation of Mo, resulting in the production of larger quantities of MoC_{1-x}-NPs.

Although some micron-sized particles were formed after the EWE process, these byproducts were filtered with cellulose and micropore filter paper (Figure S2). After this, only the α-MoC_{1-x} phase was observed in the XRD pattern (lower graph in Figure 2a). This indicates that the micron-sized byproducts were Mo and Mo_2C.

Supplementary Note 2

Evaluation of Pt content in MoC_{1-x}/Pt-600-NPs.

By ICP-AES analysis, we obtained the concentration of Mo and Pt elements in MoC_{1-x}/Pt-600-NPs (Table S3). Suppose that MoC_{1-x}/Pt-600-NPs are comprised of MoC and Pt, concentration of MoC is calculated as follows.

$$\mathbf{MoC\ concentration = Mo\ concentration} \times \frac{\mathbf{molecular\ weight\ of\ MoC}}{\mathbf{atomic\ weight\ of\ Mo}} = \mathbf{795204}$$

So, Pt content in MoC_{1-x}/Pt-600-NPs is calculated as follows.

$$\mathbf{Pt\ content(wt\%)} = \frac{\boldsymbol{Pt\ concentration}}{\boldsymbol{(MoC + Pt)\ concentration}} \times \mathbf{100 = 3.05}$$

However, because MoC_{1-x}/Pt-600-NPs contain certain amount of carbon layer, Pt content is probably lower than 3.05 wt%. Meanwhile, we investigated the SEM EDS elemental mapping of MoC_{1-x}/Pt-600-NPs in which Pt content was estimated to be 2.7 wt% (Figure S8). So, we concluded that Pt content in MoC_{1-x}/Pt-600 NPs is 2.7-3 wt%.

Supplementary Note 3

Calculation of electrochemically active surface area (ECSA).

ECSA was estimated from double-layer capacitance (C_{dl}). ECSA was calculated by dividing the C_{dl} using the specific surface capacitance (C_s) of the electrode surface:

$$\mathbf{ECSA} = \frac{\boldsymbol{C_{dl}}}{\boldsymbol{C_s}}$$

Since the exact values of C_s for all electrocatalysts are not available, a commonly used C_s value (0.04 mF cm^{-2} in alkaline media) for metal surfaces was used in this study.[S22,S23] The geometric surface area of glassy carbon electrode is 0.196 cm^2.

Reference

[S1] Y. Zhang, Y.-C. Hsieh, V. Volkov, D. Su, W. An, R. Si, Y. Zhu, P. Liu, J. X. Wang, R. R. Adzic, ACS Catal. **2014**, 4, 738.

[S2] H. Lin, Z. Shi, S. He, X. Yu, S. Wang, Q. Gao, Y. Tang, Chem. Sci. **2016**, 7, 3399-340 5.

[S3] X. Fan, Y. Liu, Z. Peng, Z. Zhang, H. Zhou, X. Zhang, B. I. Yakobson, W.A. Goddard III, R. H. Hauge, J. M. Tour, ACS Nano **2017**, 11, 384-394.

[S4] J. Wan, J. Wu, X. Gao, T. Li, Z. Hu, H. Yu, L. Huang, Adv. Funct. Mater. **2017**, 27, 1 703933.

[S5] D. Geng, X. Zhao, Z. Chen, W. Sun, W. Fu, J. Chen, W. Liu, W. Zhou, K. P. Loh. Adv. Mater. **2017**, 29, 1700072.

[S6] T. Meng, L. Zheng, J. Qin, D. Zhao, M. Cao. J. Mater. Chem. A **2017**, 5, 20228-20238.

[S7] C. Lv, Z. Huang, Q. Yang, G. Wei, Z. Chen, M. G. Humphrey, C. Zhang, J. Mater. Ch em. A **201**7, 5, 22805-22812.

[S8] J. Jia, W. Zhou, Z. Wei, T. Xiong, G. Li, L. Zhao, X. Zhang, H. Liu, J. Zhou, S. Chen, Nano Energy **2017**, 41, 749-757.

[S9] L. Ji, J. Wang, X. Teng, H. Dong, X. He, Z. Chen, ACS Appl. Mater. Interfaces **2018**, 10, 14632-14640.

[S10] J. Zhu, Y. Yao, Z. Chen, A. Zhang, M. Zhou, J. Guo, W.D. Wu, X.D. Chen, Y. Li, Z. Wu, ACS Appl. Mater. Interfaces **2018**, 10, 18761-18770.

[S11] K. Zhang, G. Zhang, J. Qu, H. Liu, ACS Appl. Mater. Interfaces **2018**, 10, 2451-2459.

[S12] J. T. Ren, L. Chen, C. C. Weng, G. G. Yuan, Z. Y. Yuan, ACS Appl. Mater. Interfaces **2018**, 10, 33276-33286.

[S13] M. A. R Anjum, M. H. Lee, J. S. Lee, ACS Catal. **2018**, 8, 8296-8305.

[S14] J. Xiong, J. Li, J. Shi, X. Zhang, N. T. Suen, Z. Liu, Y. Huang, G. Xu, W. Cai, X. Lei, L. Feng, Z. Yang, L. Huang, H. Cheng, ACS Energy Lett. **2018**, 3, 341-348.

[S15] J. Diao, W. Yuan, Y. Su, Y. Qiu, X. Guo, Adv. Mater. Interfaces **2018**, 5, 1800223.

[S16] Z. Cheng, Q. Fu, Q. Han, Y. Xiao, Y. Liang, Y. Zhao, L. Qu, Adv. Funct. Mater. **2018**, 28, 1705967.

[S17] H. Yan, Y. Xie, Y. Jiao, A. Wu, C. Tian, X. Zhang, L. Wang, H. Fu, Adv. Mater. **2018**, 30, 1704156.

[S18] X. Zang, W. Chen, X. Zou, J. N. Hohman, L. Yang, B. Li, M. Wei, C. Zhu, J. Liang, M. Sanghadasa, J. Gu, L. Lin, Adv. Mater. **2018**, 1805188.

[S19] H. Wei, Q. Xi, X. Chen, D. Guo, F. Ding, Z. Yang, S. Wang, J. Li, S. Huang, Adv. Sci. **2018**, 5, 1700733.

[S20] J. Dong, Q. Wu, C. Huang, W. Yaok Q. Xu, J. Mater. Chem. A **2018**, 6, 10028-10035.

[S21] L. Diao, J. Qin, N. Zhao, C. Shi, E. Liu, F. He, L. Ma, J. Li, C. He, J. Mater. Chem. A **2018**, 6, 6054.

[S22] C. C. L. McCrory, S, Jung, J. C. Peters, T. F. Jaramillo, J. Am. Chem. Soc. **2013**, 135, 16977– 16987.

[S23] Y. Zhang, L. Gao, E. J. M. Hensen, J. P. Hofmann, ACS Energy Lett. **2018**, 3, 1360-13 65.
